# A global assessment of actors and their roles in climate change adaptation

**DOI:** 10.1038/s41558-023-01824-z

**Published:** 2023-10-12

**Authors:** Jan Petzold, Tom Hawxwell, Kerstin Jantke, Eduardo Gonçalves Gresse, Charlotta Mirbach, Idowu Ajibade, Suruchi Bhadwal, Kathryn Bowen, Alexandra Paige Fischer, Elphin Tom Joe, Christine J. Kirchhoff, Katharine J. Mach, Diana Reckien, Alcade C. Segnon, Chandni Singh, Nicola Ulibarri, Donovan Campbell, Emilie Cremin, Leonie Färber, Greeshma Hegde, Jihye Jeong, Abraham Marshall Nunbogu, Himansu Kesari Pradhan, Lea S. Schröder, Mohammad Aminur Rahman Shah, Pauline Reese, Ferdous Sultana, Carlos Tello, Jiren Xu, The Global Adaptation Mapping Initiative Team, Jan Petzold, Tom Hawxwell, Idowu Ajibade, Suruchi Bhadwal, Kathryn Bowen, Alexandra Paige Fischer, Elphin Tom Joe, Christine J. Kirchhoff, Katharine J. Mach, Diana Reckien, Alcade C. Segnon, Chandni Singh, Nicola Ulibarri, Donovan Campbell, Emilie Cremin, Greeshma Hegde, Abraham Marshall Nunbogu, Mohammad Aminur Rahman Shah, Jiren Xu, Matthias Garschagen, Matthias Garschagen

**Affiliations:** 1 Department of Geography, Ludwig-Maximilians-Universität München, München, Germany; 2 DFG-Research Training Group ‘Urban future-making’, HafenCity University Hamburg, Hamburg, Germany; 3 Center for Earth System Research and Sustainability, University of Hamburg, Hamburg, Germany; 4 Department of Environmental Sciences, Emory University, Atlanta, GA USA; 5 The Energy and Resources Institute, New Delhi, India; 6 Melbourne Climate Futures & Melbourne School of Population and Global Health, University of Melbourne, Melbourne, Victoria Australia; 7 School for Environment and Sustainability, University of Michigan, Ann Arbor, MI USA; 8 Department of Civil & Environmental Engineering, Penn State, University Park, PA USA; 9 School of Engineering Design and Innovation, Department of Civil & Environmental Engineering, Penn State, University Park, PA USA; 10 Department of Environmental Science and Policy, Rosenstiel School of Marine, Atmospheric, and Earth Science, University of Miami, Miami, FL USA; 11 Leonard and Jayne Abess Center for Ecosystem Science and Policy, University of Miami, Coral Gables, FL USA; 12 Department of Urban and Regional Planning and Geo-Information Management, Faculty of Geo-Information Science and Earth Observation, University of Twente, Enschede, The Netherlands; 13 International Center for Tropical Agriculture (CIAT), Dakar, Senegal; 14 Faculty of Agronomic Sciences, University of Abomey-Calavi, Cotonou, Republic of Benin; 15 School of Environment and Sustainability, Indian Institute for Human Settlements, Bangalore, India; 16 Department of Urban Planning & Public Policy, University of California Irvine, Irvine, CA USA; 17 Department of Geography & Geology, University of the West Indies, Kingston, Jamaica; 18 School of Social and Environmental Sustainability and Interdisciplinary Studies, University of Glasgow, Glasgow, UK; 19 Institute of Marine Ecosystem and Fishery Science, Center for Earth System Research and Sustainability, University of Hamburg, Hamburg, Germany; 20 Maharashtra, India; 21 Research Unit Sustainability and Climate Risks, Center for Earth System Research and Sustainability, University of Hamburg, Hamburg, Germany; 22 Department of Geography and Environmental Management, University of Waterloo, Waterloo, Ontario Canada; 23 Helmholtz Zentrum Hereon, Hamburg, Germany; 24 Canadian Centre for Climate Change and Adaptation, University of Prince Edward Island, Charlottetown, Prince Edward Island Canada; 25 University of Copenhagen, Copenhagen, Denmark; 26 Research Unit Climate Change and Security, Institute of Geography, Center for Earth System Research and Sustainability, University of Hamburg, Hamburg, Germany; 27 Research Unit Critical Geographies of Global Inequalities, Institute of Geography, Center for Earth System Research and Sustainability, University of Hamburg, Hamburg, Germany; 28 School of Social and Environmental Sustainability, University of Glasgow, Dumfries, UK; 29 water@leeds, School of Geography, University of Leeds, Leeds, UK

**Keywords:** Governance, Governance

## Abstract

An assessment of the global progress in climate change adaptation is urgently needed. Despite a rising awareness that adaptation should involve diverse societal actors and a shared sense of responsibility, little is known about the types of actors, such as state and non-state, and their roles in different types of adaptation responses as well as in different regions. Based on a large *n*-structured analysis of case studies, we show that, although individuals or households are the most prominent actors implementing adaptation, they are the least involved in institutional responses, particularly in the global south. Governments are most often involved in planning and civil society in coordinating responses. Adaptation of individuals or households is documented especially in rural areas, and governments in urban areas. Overall, understanding of institutional, multi-actor and transformational adaptation is still limited. These findings contribute to debates around ‘social contracts’ for adaptation, that is, an agreement on the distribution of roles and responsibilities, and inform future adaptation governance.

## Abstract (web summary)

For global adaptation effort, it is essential to understand which actors are participating and what their roles are. This Analysis, based on comparative case studies, displays the dominant actors in adaptation, and how the actor–role patterns vary across regions.

## Main

The realization that climate change adaptation is urgent has entered mainstream planning and policymaking, and people and institutions are adapting^1–3^. The recent *Sixth Assessment Report* of the Intergovernmental Panel on Climate Change (IPCC) stresses the need to identify “who needs to take what actions and when in order that transformations unfold at sufficient speed and scale to meet the Paris, SDG and other policy goals”^4^. Societies across the globe are struggling to find and negotiate an effective and fair distribution of climate actions between state actors, civil society, citizens, the private sector and other actors. With a growing realization of the rapidly increasing adaptation tasks ahead of societies, social contracts for climate change adaptation are increasingly debated locally and globally^5^. An important starting point for such debates, however, is the empirical observation of current roles in adaptation.

While climate change adaptation is understood to be place-based, the roles and responsibility for action across actors, scales and diverse geographies are often unclear from both empirical and normative perspectives, that is, ‘who is acting how’ and ‘who should act how’, respectively^5–8^. In addition, adaptation usually occurs in multi-actor settings, where different actors play different roles. While state actors, for instance, are typically expected to act on public infrastructure or moderate institutional adaptation in the interest of society at large, private actors usually are not met with such expectations. They might act rather to their immediate benefit. Yet, depending on cultural and institutional arrangements, the boundaries between public and private actors or between different levels of government can be unclear^9,10^.

The constellation of actors, institutional arrangements and policy instruments characterizing climate change adaptation governance in a given location is highly context-specific^8,11^. Institutional and technological capacities and socioeconomic characteristics may differ considerably between urban and rural areas, as well as between the global south and global north^1,12,13^. While city governments with more resources and institutional capacities, especially those in the global north, can be, in principle, active and interconnected frontrunners in climate change adaptation planning and implementation, adaptation in many urban areas of the global south often lack such resources and capacities, and local adaptation often involves mainly informal activities^14^. Rural areas, on the other hand, predominantly in the global south, are often characterized by high degrees of poverty, limited infrastructure, a strong focus on agriculture and a degree of neglect by national policymakers. However, rural communities and actors draw on rich experience and local knowledge to cope with environmental and climatic hazards^15^.

When considering the diverse actors across geographic contexts as agents who conceive and implement adaptation rather than as mere entities exposed to climate change impacts^16^, it becomes necessary to consider the range of roles these actors play. The term role here refers to an actor’s general position or function within a larger social system and in a certain process, here, climate change adaptation. Roles come with responsibilities in terms of specific tasks and duties. On both levels, there might be differences between how actors are acting and how they should act, for example, in relation to what other actors expect from them. For the context studied here, roles can be linked to the adaptation as well as the policy cycle. The former includes assessing impacts, vulnerability, risks and resilience; planning adaptation; implementing adaptation measures; and monitoring and evaluating adaptation^17^. The latter would suggest defining roles in relation to, for example, agenda setting, awareness raising, initiating policy, coordinating interaction, target setting, strategy making, financing, enforcement and policy adjustment (see Methods for a description of the roles used in this analysis)^7^. For each of these roles, different types of actors are typically associated with specific capacities, expectations and mandates, which, however, can be unclear, overlapping or only just emerging.

Findings from the Global Adaptation Mapping Initiative (GAMI)—the first global systematic mapping of peer-reviewed literature on climate change adaptation—indicate a fragmentation among actors and interventions in the context of climate change adaptation and little evidence of transformational adaptation, which refers to adaptation with deep systemic shifts^1^. Such shifts will only be possible with a clear distribution of roles and responsibilities. All actors need to come together here with ideally a shared agreement on mutual roles^5^. Generally, households are reported more often in climate change adaptation research than government actors, but relative prominence varies across global contexts^1^. Additionally, governments tend to prioritize other adaptation interventions than businesses or civil society organizations, often based on their ability to create regulatory or market-based adaptation interventions^8^. Other reviews on actors in adaptation demonstrate unclear divisions and diverging perceptions of responsibilities between actor groups and across governance scales, which may result in barriers to the effective implementation of responses at the local scale^5,18,19^. Overcoming such barriers, for example, through more polycentric climate governance systems that bridge adaptation actions and agendas across stakeholders and scales, is crucial for enabling the multi-actor coordination required for transformational adaptation^20,21^.

While GAMI provides a rough global overview of documented actors and types of adaptation, here we aim to contribute to a deeper understanding of the types of roles specific actors play, in how far multi-actor collaboration is documented and how actor–role patterns differ across geographical contexts, including urban and rural settlements, in order to support further research on normative social contracts for adaptation and the barriers and drivers of transformational adaptation^22–25^. We ask the following questions: which roles do particular actors currently play in climate change adaptation? Which actors interact or collaborate in specific adaptation practices? To what extent can geographical patterns be identified in relation to specific actor types? How are different climate-related responses associated with specific actor types? To what extent are specific actor types associated with more transformational forms of adaptation?

To find answers to these questions, we built on GAMI’s global stocktake of human adaptation-related responses to climate-related change by re-coding the GAMI database according to actors, roles and settlement types, and by analysing the data through descriptive statistics and regression analysis. GAMI is a spatially explicit dataset derived from coding empirical studies on climate change conducted between 2013 and 2019 (ref. ^1^). A team of 21 researchers systematically re-screened and re-coded all 1,682 articles included in the GAMI database according to our specific inclusion criteria (see Methods and Supplementary Fig. 1). The results of the remaining 1,472 articles were synthesized and interpreted by 17 researchers. While the GAMI database only includes published academic literature on empirically observed adaptations, we argue that such analysis is nevertheless highly relevant as it allows us to examine how adaptation roles are treated in research—and which blind spots might exist. With rapidly expanding adaptation scholarship, literature-based studies such as this analysis can serve as a relevant information source for assessing actual adaptation on the ground, combined with a triangulation with other datasets to address potential language bias and other difficult-to-quantify selection biases and gaps in the academic literature.

## Actors and roles in adaptation

**Fig. 1:**
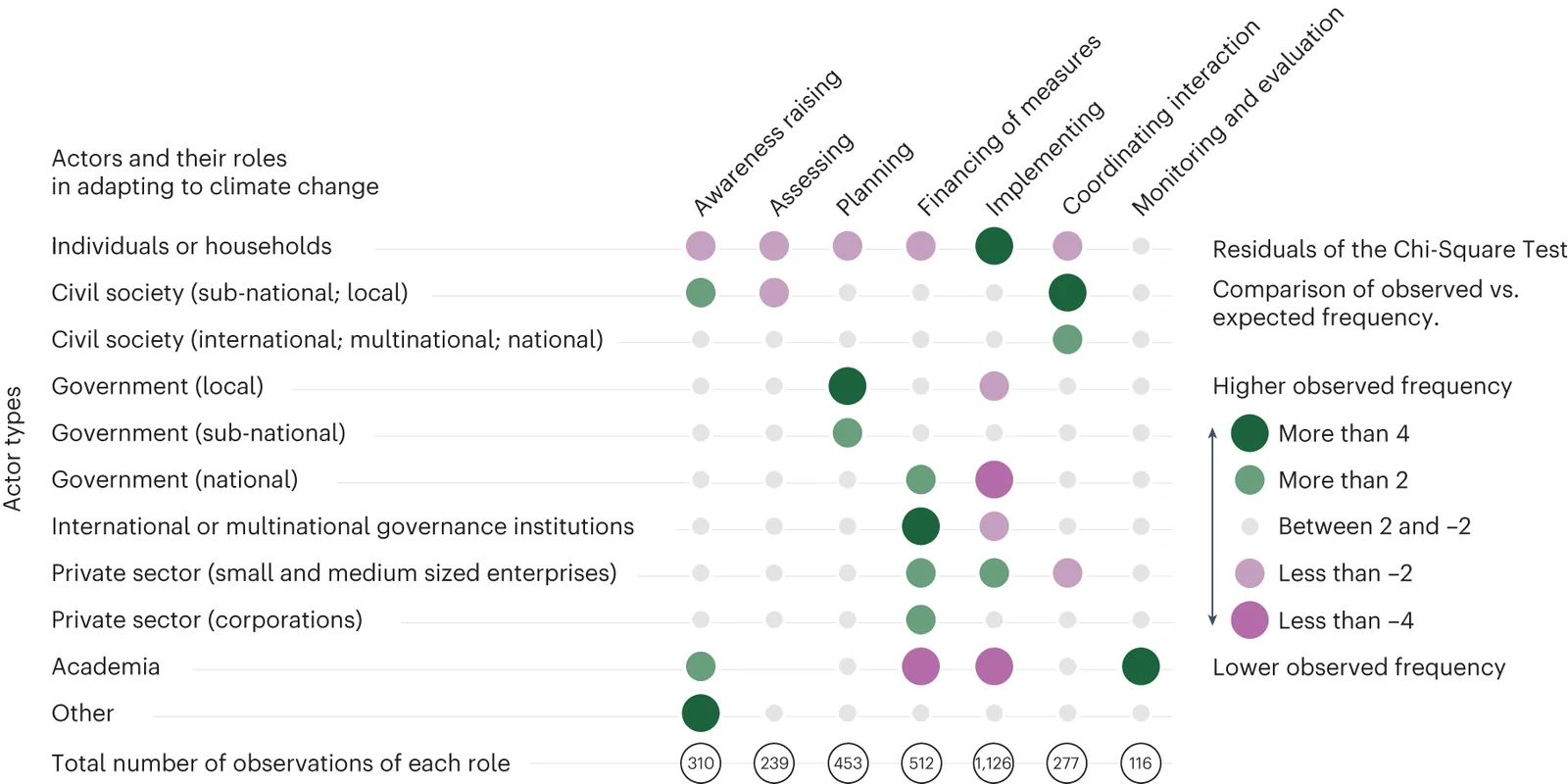
Actors and their roles in adapting to climate change. Results of the chi-square test calculating the residuals, that is, the difference between the observed and the expected frequency of each combination of actor and adaptation role, are shown. Residuals below −2 and above 2 can be considered as contributing most to the significant result (*P* < 0.001) of the chi-square test. Positive residuals (green) indicate a higher observed frequency of an actor–role combination, and negative residuals (purple) indicate a lower-than-expected frequency. The size of the circles corresponds to the value of the residuals. *χ*^2^ = 610.77, d.f. = 70, *P* < 0.001. See Supplementary Table 2 for a detailed overview of the results. Source data

Our findings reveal several patterns in which actors were more likely than others to take on specific adaptation roles. Overall, individuals or households are the most frequently reported actor type (representing 64% of coded articles) and by far the most documented actors for the actual implementation of adaptation measures (Fig. 1). The role of financing is mainly associated with international or multinational governance institutions, the private sector, civil society organizations and national governments. Planning is more often done by government actors, with no particular pattern discernible at different government levels. Civil society organizations are important actors for coordinating the interaction between various other actors and raising awareness. Awareness raising is also a relevant role associated with academia, along with assessing climate impacts and monitoring adaptation efforts. While there is little reporting on private sector actors across all regions, their documented roles are mainly related to financing and implementing adaptation responses.

## Multi-actor constellations in adaptation

**Fig. 2:**
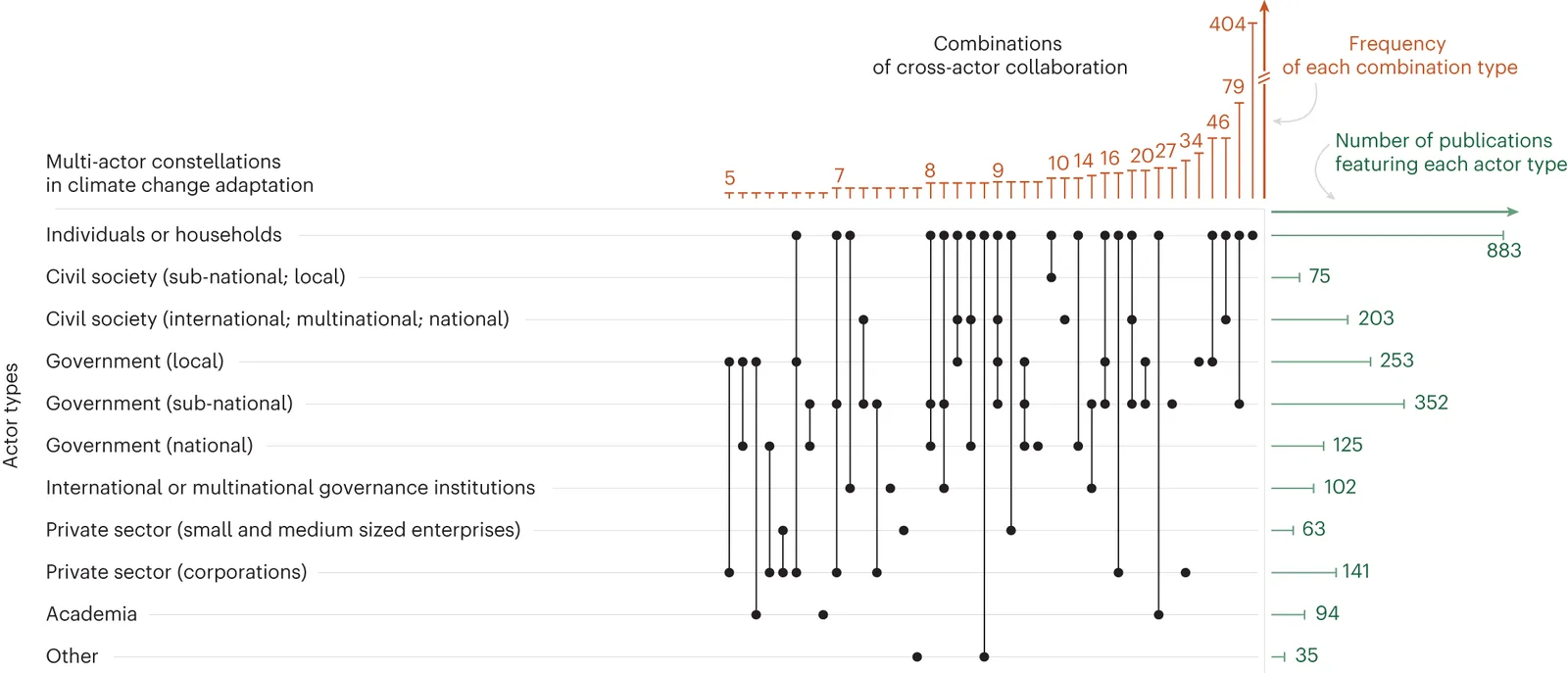
Multi-actor constellations in adaptation. Frequency of actor types reported (green horizontal bars), as well as how often they were reported as single actor type in a study (single dots and red vertical bars) or in combination with other actor types (connected dots and red vertical bars). Source data

In almost a third of the coded articles (404 articles), individuals or households are the sole reported actor type (Fig. 2). The most common combination of actors is individuals or households with national governments (79 articles). The second most common combination of actor types is individuals or households with local government or sub-national civil society (46 articles each). There is limited evidence of cases involving national and local governments and constellations with diverse actor types (for example, constellations involving governance actors together with the private sector or civil society actors and individuals or households). The most common example of such a constellation links individuals or households with local civil society and the national government (17 articles). There is also little reported evidence of small and medium-sized enterprises (SMEs) collaborating with government actors (seven articles with SMEs and national government; six articles with SMEs and local government). Although some examples of activities involve private households and SMEs (16 articles), reports of SMEs and other actor types are rare. Actors from academia mainly appear in collaboration with individuals or households (20 articles) and local government (five articles).

## Urban–rural actor patterns

**Fig. 3:**
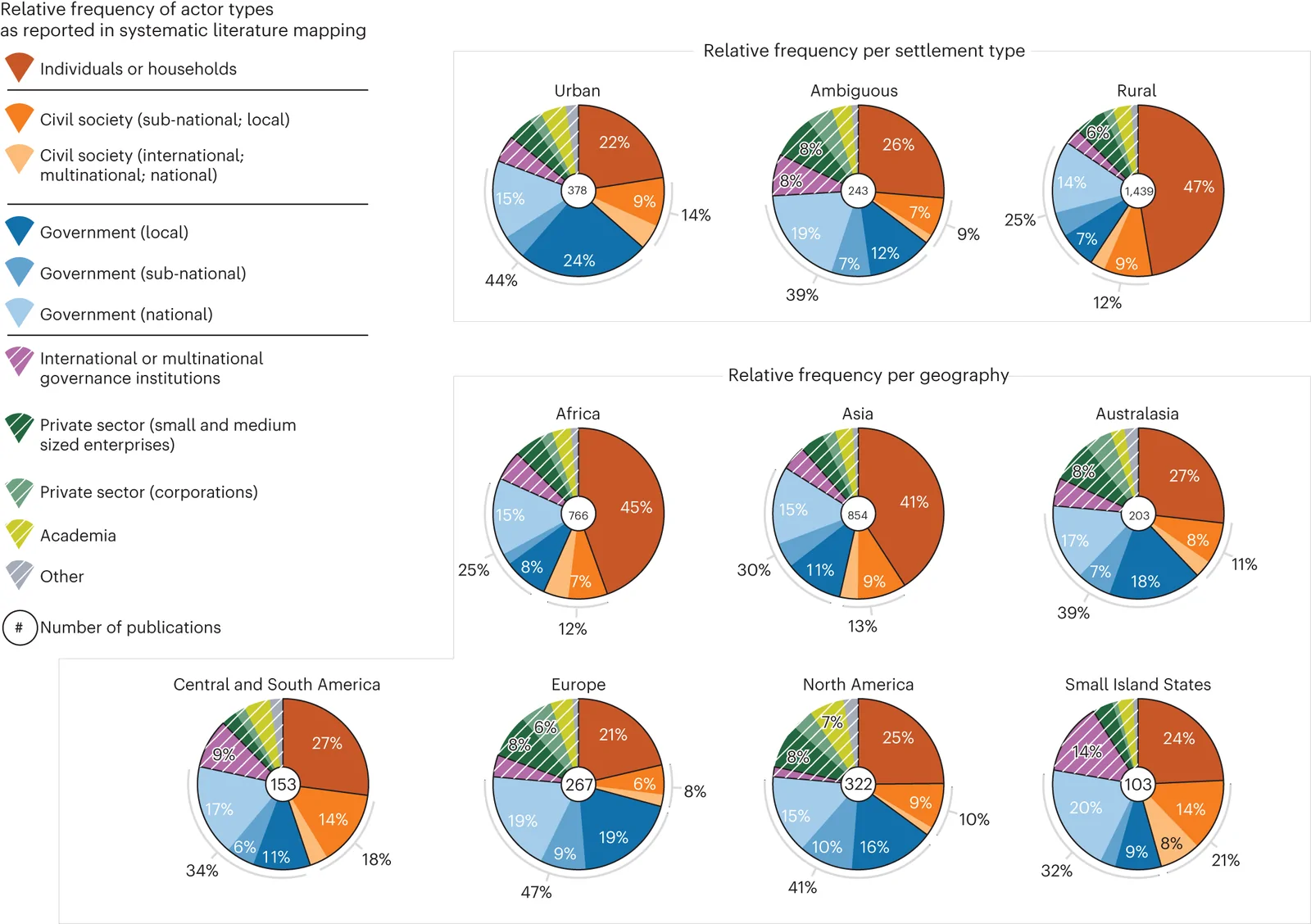
Regional and urban–rural actor patterns in adaptation. Relative frequency of actor types reported in publications per settlement type and region. Source data

The majority of the publications (65%) report adaptation in rural areas. Here, individuals or households are by far the most frequently reported actors (47%; Fig. 3). In comparison, government actors (from local to national levels) make for a much smaller fraction of the reported actors (together 25%). In contrast, the picture is almost inverse in urban settings. Here, government actors (across levels) account for 44% of the reported actors, while individuals or households contribute only 22%. In addition, national governments and sub-national civil society actors are reported more often than local governments in rural contexts, while in urban areas, the focus is on local government actors. There is little difference between urban and rural case studies for other actor types. In ambiguous studies—those with mid-sized populations that are neither clearly urban nor rural or mixed-case studies—individuals or households are reported most often (26%), followed by national and local governments.

## Actors by region

The distribution of actors reported in adaptation studies varies across regions. Individuals or households are the most frequently mentioned actors in all regions (Fig. 3). In Africa, Asia, Central and South America, and the Small Island States, national governments are the second most common actor type. In Europe, national and local governments are at the same ratio (both 19%), and in North America and Australasia, local governments are mentioned slightly more often. However, when considering all three levels of government actors in combination (that is, local, sub-national and national level), a clear pattern emerges: in higher-income regions, particularly Europe, North America and Australasia, government actors represent the largest group of reported adaptation actors (that is, larger than individuals/households). In contrast, individuals/households are reported much more often than all government actors combined in Asia and particularly in Africa. Civil society actors are reported particularly frequently in Central and South America and the Small Island States. In the Small Island States, international or multinational governance institutions are also reported frequently (14% of the cases, the largest fraction across all regions). The global scale rural-to-urban differences between government actors and individuals or households (see ‘Urban–rural actor patterns’) are even more pronounced when considering different world regions in the global north and global south. Government actors are dominantly represented in urban areas, particularly strongly in Australasia, Europe and North America (see Supplementary Fig. 3). For rural areas, regional differences are smaller but individuals or households are still relatively more represented in Africa and Asia than in other world regions.

## Actor roles by region

In terms of actor roles, we observed some commonalities but also differences across world regions. Individuals or households are by far the single most reported actors for implementing adaptation measures in all world regions. In all regions, government actors are mainly involved in planning and implementation processes. Especially in Europe, local governments are more associated with planning than in other regions. In North America, sub-national governments play a greater role in planning, implementing, monitoring and evaluating compared with other regions. In contrast, in Africa, sub-national governments are less involved in planning (Table 1 and Supplementary Table 3).

**Table 1:** Patterns in actor–role combinations across world regions

Region	Actor–role combination	Residuals	Observations	Percentage of regional observations
Africa	Government (sub-national) + Planning	−4.09	2	0.16
Africa	Individuals or households + Implementing	7.87	333	26.51
Asia	Individuals or households + Implementing	4.27	337	22.63
Europe	Government (local) + Planning	4.39	40	7.34
Europe	Individuals or households + Implementing	−5.76	54	9.91
North America	Government (sub-national) + Implementing	4.90	24	3.65
North America	Government (sub-national) + Monitoring and evaluating	4.51	7	1.07
North America	Government (sub-national) + Planning	4.38	20	3.04
North America	Individuals or households + Implementing	−5.13	77	11.72
North America	Other + Coordinating interaction	4.32	4	0.61

Results of the chi-square test calculating the residuals, that is, the differences between the observed and the expected frequency of each combination of actor and adaptation role per region. Here all results of combinations with residuals below −2 and above 2 are shown. Residuals below −2 and above 2 can be considered as contributing most to the significant result (*P* < 0.001) of the chi-square test. See Supplementary Table 3 for the full list of test results.

## Nature of responses by actors

**Fig. 4:**
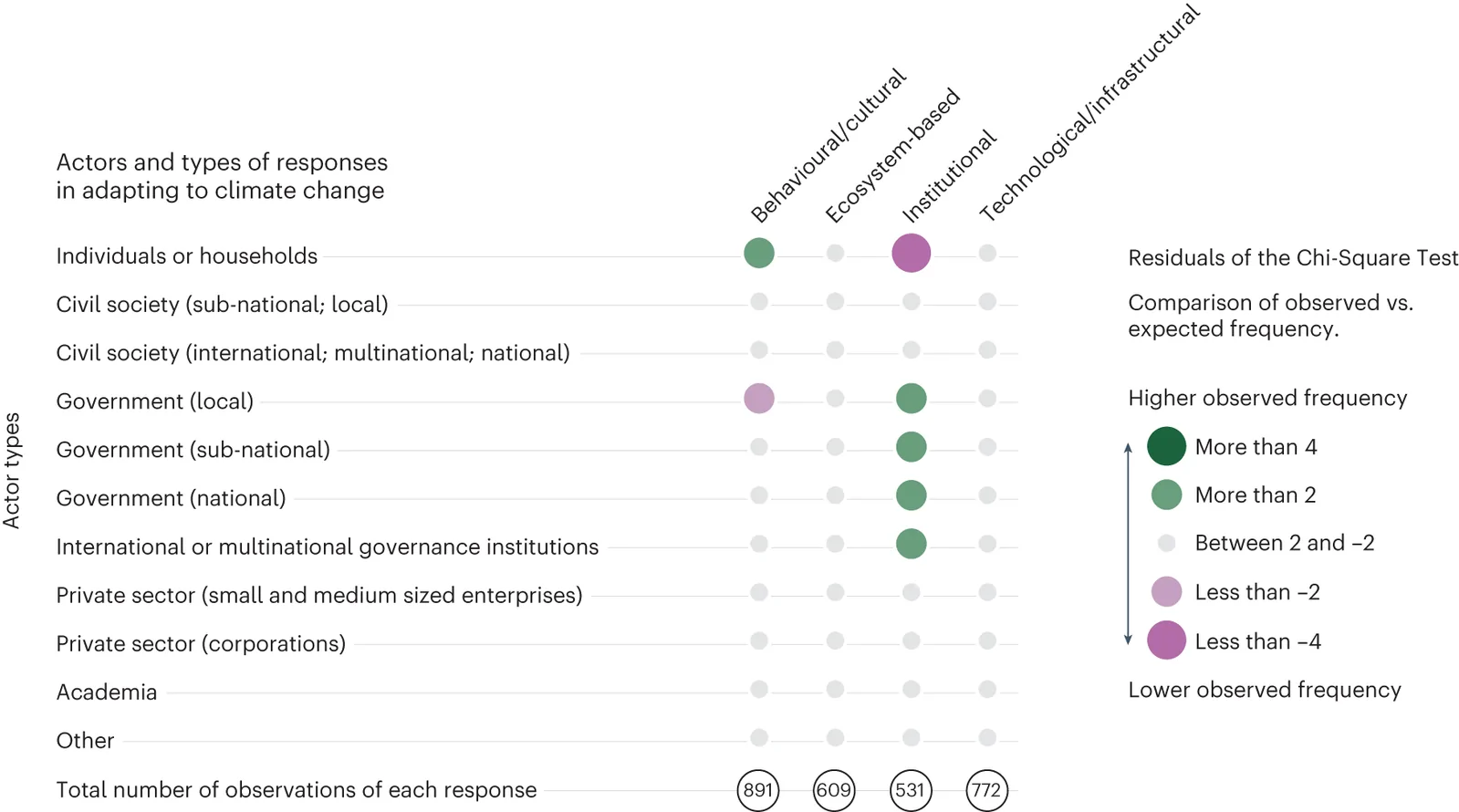
Actor types per response type. Results of the chi-square test calculating the residuals, that is, the difference between the observed and the expected frequency of each combination of actor and response type. Residuals below −2 and above 2 can be considered as contributing most to the significant result (*P* < 0.001) of the chi-square test. Positive residuals (green) indicate a higher observed frequency of a respective actor–response type combination, and negative residuals (purple) indicate a lower frequency than expected. The size of the circles corresponds to the value of the residuals. *χ*^2^ = 87.552, d.f. = 30, *P* < 0.001. Source data

Except for governments or international governance institutions, behavioural/cultural responses are the most common response of all actor types. However, there are differences in the relative association of actors and responses (Fig. 4). Individuals and households are mainly associated with behavioural/cultural and less with institutional responses. For government actors, including international and multinational governance institutions, institutional responses are most common.

Overall, there is limited evidence in the GAMI database of more transformational forms of adaptation that include deep systemic shifts. All actor types are most often associated with low-depth adaptations, that is, responses characterized by a continuation of existing practices or only small changes. The analysis of how responses of high depth are characterized concerning actor types, roles, response types, regions and settlement types reveals that they are frequently associated with case studies in Small Island States (region), institutional adaptation (response type), international governance institutions (actor) and the coordination of adaptation (role). They are less likely to be associated with studies reporting on behavioural/cultural responses and the role of monitoring and evaluating (Table 2).

**Table 2:** Characteristics of medium- to high-depth responses in relation to the variables actor type, role, response type, region and settlement type

	Ordered regression coefficient	s.e.m.	*P* value
Small Island States	1.308	0.401	0.001
Behavioural/cultural	−0.651	0.180	0.000
Institutional	0.553	0.161	0.001
International or multinational governance institutions	0.569	0.269	0.034
Coordinating interaction	0.392	0.185	0.034
Monitoring and evaluating	−0.736	0.260	0.005

Statistically significant (*P* < 0.05) coefficients of the ordered logistic regression predict depth levels. Coefficients are the log odds of low versus medium depth or medium versus high depth when a given variable exists. s.e.m., standard error of the mean. Studies that mention Small Island States, for instance, have a 3.7 (exp(1.3) = 3.7) times higher likelihood of being associated with higher depth levels than studies that do not mention Small Island States. See Supplementary Table 5 for the full list of results.

## Discussion

The analysis of climate change adaptation documented in the scientific literature reveals important patterns regarding actors and the roles they perform. Individuals and households are the actors reported to be implementing adaptation the most. Government actors, on the other hand, are reported to be predominantly involved in planning and funding adaptation. The strong evidence for individuals or households as prime actors for the implementation of actual adaptation aligns with earlier research concluding that adaptation is often a highly localized phenomenon with a tendency towards autonomous and incremental adaptation^26–28^. This pattern is further supported by our finding that individuals and households are mostly engaged with behavioural/cultural responses. The relatively low representation of governments in the actual implementation of adaptation may indicate an implementation gap. Governments are the actors to ratify political agendas and agreements for climate action (from the global to the local level) and could be expected to also take a leading role in the implementation of actual adaptation projects. On the other hand, this low representation might partly be due to the fact that some forms of government action contributing towards adaptation might not be labelled as such in the literature, such as general poverty alleviation initiatives, social security programmes, infrastructure development and education.

The assessment further revealed evidence suggesting that in terms of response types, individuals and households play only a limited role in institutional adaptation. Institutional responses may, by definition, be driven by government or civil society actors. However, in order to facilitate the transformation of institutions in a participatory and inclusive manner, individuals or households are key actors, and their active engagement has been argued to be essential for the success of such processes^29^. More research on the role of these actors in institutional adaptation is therefore needed. In addition, our findings yield quite a striking picture regarding actors involved in ecosystem-based and technological/infrastructure adaptation. While the literature stresses that successful ecosystem-based adaptation relies on the active involvement of local communities^30,31^, we did not find that a particularly strong involvement of households or individuals, local civil society actors or local governments has been reported in the literature. Similarly, we did not find a particularly strong involvement of government actors or the private sector in technological/infrastructure adaptation, despite the fact that both actor types could be expected to play such a role. Both of these findings clearly warrant further research and triangulation with other data sources.

Our findings also identify actors hitherto underrepresented in climate change adaptation research, most notably the private sector. This confirms other lines of evidence suggesting that the private sector lags behind in implementing adaptation^32–34^. This lag is surprising, given not only the increasing impacts of climate change on the private sector but also the central role of the private sector in shaping overall development trends—and hence successful climate change adaptation at large. Exploring the existing and potential roles of private sector actors in adaptation, particularly in financing and implementation processes and in contexts where market-based interventions are promoted, is therefore needed to advance our understanding of the enablers, barriers and responsibility shifts in climate change adaptation overall^35,36^. Academia is most prominently documented as being involved in monitoring and evaluation, which is a role that is overall not widely covered in the adaptation activities reported in the academic literature. Hence, there is a general need for further academic attention to monitoring and evaluation, and, in addition, to the capacities of different actor types beyond academia to fulfil this role. A better understanding of actor roles and capacities for monitoring and evaluation can also help streamline methods for evaluating the effectiveness of implemented adaptation measures and avoid maladaptation^37^.

While adaptation is generally considered a multi-actor process^4^, there is limited evidence of collaboration between more than two different actor types (for example, individuals or households along with civil society and government actors) and, therefore, limited empirical understanding of how such multi-actor processes can support effective adaptation. The relative prominence of local civil society actors in coordinating climate-related responses indicates how a specific actor might find a specific role in adaptation. Moreover, our findings confirm that adaptation in climate change hotspots, for example, Small Island States, is often associated with potentially transformational responses, such as relocation. Such kinds of responses result in a particularly strong need for collaboration because they involve different actors and come with a high potential for conflicting interests that need to be mediated^38–40^. At the same time, the dominance of adaptation studies with a single-actor focus raises the question of whether this dominance is due to a research bias towards individual actor types or relates to a lack of cooperation on the ground. Future research should thus more explicitly explore the relationships between actors and their roles in multi-actor constellations.

Our actor mapping further shows that it is important to consider differences between settlement types when empirically assessing and normatively debating the roles and responsibilities of actors in adaptation. We found that the majority of reported adaptation takes place in rural areas, which, however, may to a certain extent reflect a research bias on adaptation in resource-dependent communities that focus on households and on-the-ground responses to climate-related changes, for example, within the research on community-based adaptation^41^. However, with increasing urbanization and tangible impacts on urban populations, the research focus is already shifting towards urban areas^14,42^. Our finding that government actors—particularly local governments—play a less prominent role in rural areas than in urban areas may reflect that rural areas are often outside municipal jurisdictions, and that agricultural and rural development policies are often centralized nationally, with little regional autonomy. At the same time, the stronger role of government actors in urban adaptation might be driven by the fact that urban adaptation is, to a large part, concerned with the adjustment of infrastructure, much of it public. In terms of reported case studies, we found most rural cases situated in the global south, that is, in regions where the effects of climate change are already clearly felt and, thus, adaptation responses seem particularly relevant^1,43^. In addition to the differences between settlement types, we found strong regional differences regarding actor roles. Overall, government actors are more often documented in the global north, and individuals or households more prominently in the global south. This finding suggests that there are currently differences in the progress of government-led and planned adaptation, which highlights the relevance of regionally sensitive adaptation tracking (for example, within the context of the global stocktake) and context-specific debates regarding the distribution of responsibilities for equitable and effective adaptation action^18^.

In the global picture, there is limited evidence of transformational adaptation^1^. Further research on actors and implementation gaps in the context of climate change adaptation may shed light on the barriers and potential drivers of transformational adaptation. As different normative views of adaptation processes as well as outcomes, arising from different epistemological and disciplinary entry points, can lead to different interpretations of adaptation effectiveness, future research needs to further explore how different actors frame adaptation effectiveness^44,45^. Our findings reflect an overall rather incremental implementation of adaptation, where individual and household adaptation responses are mainly behavioural, low in depth and with only a limited connection to institutional change, while government actors are in many contexts less prominent, particularly in implementing adaptation. Such incremental adaptation is important for local communities in dealing with climate risks. However, our findings suggest a lack of preconditions for more transformational adaptation, which requires systemic change and multi-actor collaboration^46^, building on broad societal support and a shared agreement on roles and responsibilities: a social contract for climate action and sustainability transformations.

## Methods

We built on GAMI’s systematic literature mapping, which provides a global stocktake of human adaptation-related responses to climate-related changes in human systems^1^. Combining a novel approach that integrates systematic reviewing and machine learning, diverse databases were searched, including the Web of Science, Scopus, PubMed and Google Scholar, to assess 48,316 scientific documents on adaptation published in peer-reviewed literature between 2013 and 2019. With this method, only evidence on adaptation documented in this body of literature is included, and other forms of literature (for example, grey literature) are excluded. After the screening was done, based on the applied inclusion criteria, the data corpus was narrowed down to 1,682 publications relevant to human adaptation responses to climate change^1^.

### Screening

To explore the types of actors and their roles in climate change adaptation in greater detail, we conducted a further screening and coding of the GAMI literature. In the screening step, we filtered for articles with sufficient empirical information about the actors and/or the roles in the observed adaptation case studies and for articles of which full texts were available. Out of the 1,682 articles in the GAMI literature corpus, we selected 1,472 in this step (see Supplementary Fig. 1). Our coding scheme included two main categories—actors and roles. The development of the coding scheme was inspired by a qualitative review approach^47,48^ whereby at least two independent reviewers read a sample of the full texts and generated codes in response to the research questions independently. The authors then generated broad categories internally and applied the emergent framework to the remaining data corpus while returning to previously coded documents to update the coding to reflect newly developed categories.

### Coding

The categorization of ‘actors’ in climate change adaptation built closely on the GAMI methodology to provide the highest possible consistency. In the GAMI database, actors in climate change adaptation are categorized into nine groups (organized by sector and level): individuals or households; civil society (sub-national or local; international, multinational or national); government (local; sub-national; national); international or multinational governance institutions; and private sector (SMEs, corporations).

During a test coding phase in the development of the coding scheme, we coded a sample of the GAMI literature to identify whether additional codes should be added. In several case studies from the GAMI database, academic institutions were reported as having an explicit role in contributing to local climate change adaptation initiatives. Therefore, we added ‘academia’ as a specific actor type. The definition of an actor for the coding is “a social entity, that is, a person or organization, or a collective of persons and organizations, which is able to act”^49^. Moreover, we focused on ‘operators’ exercising adaptation rather than ‘receptors’ of adaptation or ‘exposure units’, while acknowledging that these might overlap^16^.

Roles in adaptation—the key aspect of this review—were not coded in GAMI. Articles in GAMI provide extreme diversity in the degree of detail provided for particular actors and potential roles. We started with a broad categorization of stages in the adaptation process^17^, on the one hand, and a particularly detailed conceptualization of roles and responsibilities in adaptation^7^, on the other hand. This process resulted in a coding framework with seven roles. We distinguished the roles of awareness raising, assessing, planning, financing, implementing, coordinating interaction, and monitoring and evaluating as relevant phases in climate change adaptation that may build on each other (see Supplementary Fig. 2). However, the respective roles do not always follow the sequence shown, but may also overlap or are performed in parallel by different actors. For example, financing and coordinating interactions were found to be important foundations for any action, but can also run throughout the phases.

While the GAMI database provides information about regions and sectors, including evidence from urban and rural areas, it does not systematically distinguish between these settlement types. As the roles of different actors may differ greatly in urban and rural areas, we added an additional code for settlement type, distinguishing urban, rural and ambiguous settlements^50^.

Two independent reviewers screened and coded each article to minimize bias, using the online platform Sysrev^51^. A third reviewer resolved conflicting codes. After these were resolved, the new dataset was merged with the GAMI dataset. To address the question regarding regional patterns, we used the GAMI category ‘geography’, which categorizes the papers according to the IPCC regions. Regarding different types of responses, we used the GAMI categories, which distinguish behavioural/cultural, ecosystem-based, institutional and technological/infrastructural responses.

To address the question of potentially transformational adaptation, GAMI uses the four dimensions ‘depth’, ‘scope’, ‘speed’ and ‘limits’. For the analysis in this review, we adopted the GAMI coding of the ‘depth’ category, which we consider most relevant for the purpose of our review of transformational adaptation. GAMI categorizes the depth of observed adaptations as involving a low, medium or high level of change. A high level of depth can serve as an indicator for transformational adaptation because it “reflect(s) entirely new practices that involve deep structural reform, complete changes in mindset, major shifts in perceptions or values, and changing institutional or behavioural norms”^1^ (see Supplementary Table 4 for further explanation of the coding for ‘depth’).

### Analysis

All analyses were performed using R statistical software and Python^52^. We merged the GAMI database with our additional coding regarding actors and roles. This resulted in a data frame that contains various categorical variables, for which we calculated descriptive statistics. First, we cross-tabulated each actor type with the role, settlement, region, response and depth variables. In cases where records mentioned more than one category per variable (such as more than one actor type), the record was treated as multiple observations, with each given a new row in the data frame. We performed the chi-square test of independence to determine whether certain variable combinations occur more or less frequently than expected if the null hypothesis were correct. We calculated the residuals to identify which category combinations make the greatest contribution to the chi-square test results. The tests were performed for various variable combinations, such as actor–actor combinations, actor–role combinations and actor–role combinations between regions.

We conducted an ordered logistic regression using R’s MASS package to understand variables associated with higher depth levels^53,54^. We specifically included the variables geography, response types, adaptation roles, actors and settlement type as predictors for depth levels. All categorical predictor variables were dummy-encoded as binary variables to allow for ordered logistic regression, and the response variable (depth) was encoded as an ordered factor containing the levels ‘low’, ‘medium’ and ‘high’.

### Limitations

The GAMI database only considers evidence from responses documented in peer-reviewed literature and excludes mere commitments, strategies or visions. Many on-the-ground responses to climate-related hazards may not be included in peer-reviewed literature but documented in other forms of literature (that is, grey literature), for example, reports by the private sector or civil society actors. Similarly, GAMI is strongly biased towards English-language literature due to the search string, which excludes evidence published in other languages and, thus, potentially implies an underrepresentation of evidence on adaptation from non-English speaking countries. Including languages other than English would therefore be a valuable step in future projects mapping global adaptation^55,56^.

## Online content

Any methods, additional references, Nature Portfolio reporting summaries, source data, extended data, supplementary information, acknowledgements, peer review information; details of author contributions and competing interests; and statements of data and code availability are available at https://doi.org/10.1038/s41558-023-01824-z.

## Supplementary information

## Source data

## Data Availability

All data generated or analysed during this study are included in this published article (and its supplementary information files). Source data are provided with this paper.

## Code availability

The code used in this analysis is available at: https://doi.org/10.5281/zenodo.7524551.
