# Supplementary material for: A global assessment of actors and their roles in climate change adaptation
Source: Nat Clim Chang. 2023 Oct 12;13(11):1250–7. doi: 10.1038/s41558-023-01824-z (PMC13538072; doi:10.1038/s41558-023-01824-z)
Supplement: Supplementary file 1 [file 41558_2023_1824_MOESM1_ESM.pdf]

nature climate change

Analysis

https://doi.org/10.1038/s41558-023-01824-z

A global assessment of actors and their roles in climate change adaptation

In the format provided by the authors and unedited

Supplementary Information

This supplementary material contains the following information:

1 Screening flowchart
2 Coding framework
3 Actor-roles analysis
4 Actor-roles analysis per region and settlement type
5 Transformational adaptation analysis
6 References

1. Screening flowchart

Figure 1 illustrates the screening process, building on the GAMI database, and applying our specific inclusion criteria relating to sufficiency in information about actors involved in climate-related responses and their roles. Climate-related responses need to be documented/observed and published in peer-reviewed literature.

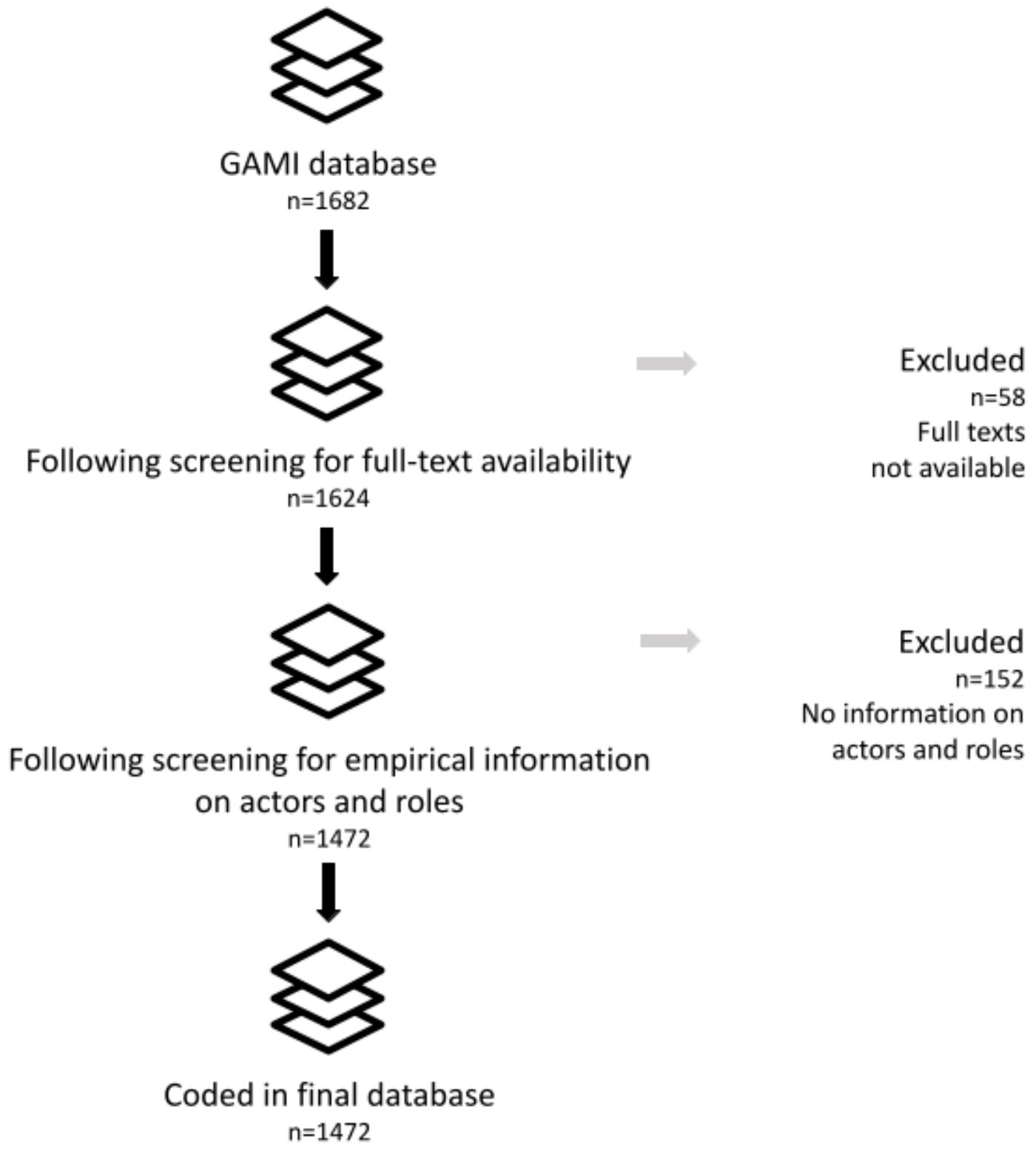

Figure 1: Selection of articles to be reviewed/coded on actors and roles in climate change adaptation.

2. Coding framework

Figure 2 illustrates the adaptation cycle and the set of actors involved in its various stages. Table 1 explains the specific variables, labels and definitions used for the additional coding building on the GAMI dataset.

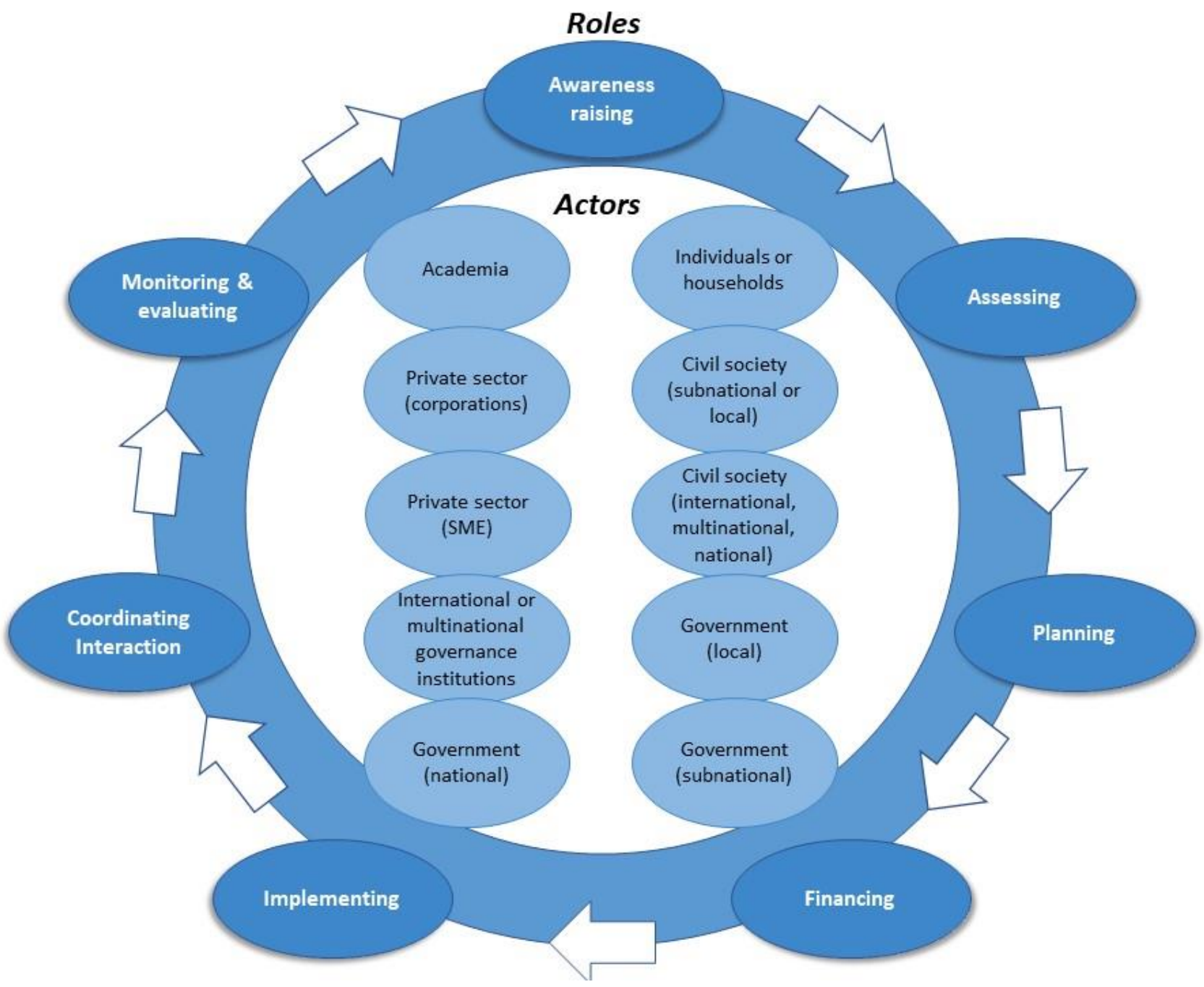

Figure 2: Actors and roles in the process of climate change adaptation. The roles occur in an iterative and timely overlapping way. Actors can be involved in one or more of the various adaptation roles.

Table 1: Coding framework

Variable	Label	Definition/example
Settlement type[1,2]	Urban	Unambiguously urban centres with much of the economically active population deriving their living from manufacturing or services. In virtually all nations, settlements with 20,000+ inhabitants are considered as urban
	Rural	Unambiguously rural settlements with most of the inhabitants deriving a living from farming and/or forestry or fishing. Populations of rural settlements range from farmsteads to a few hundred inhabitants.
	Ambiguous	‘Large villages’, ‘small towns’ and ‘small urban centres’. The proportion of the population in rural and urban areas is influenced by each nation’s definition of ‘urban areas’. Populations range from a few hundred to 20,000 inhabitants.
Actor type[3]	Individuals or households	Including informal community networks
	Civil society (sub-national or local)	Formal community associations
	Civil society (international/multinational/national)	Voluntary civil society organizations. Includes charities, non-profits, faith-based organizations, professional organizations (e.g. labour unions, associations, federations), cultural groups, religious groups, sporting associations, advocacy groups (e.g. NGOs).
	Government (local)	Terms include municipality, local government, community, urban, urban regions, rural, etc.
	Government (sub-national)	Domestic, sub-national governing unit. Terms include state, province, territory, department, canton, Bundesländer, etc.

	Government (national)	Countries officially recognized by the UN
	International or multinational governance institutions	Global or regional treaty body or agency (e.g. UN institutions/organizations, EU instittutions, Organization of American States, African Union)
	Private sector (SME)	Small- and medium-enterprises
	Private sector (corporations)	Large national or international companies
	Academia	Academic institutions such as private or public research institutes, think tanks, or universities
	Other	Any other actor type
Role[4,5]	Awareness raising	Convincing politicians of the need to do adaptation planning and /or to integrate adaptation in other policy areas. Or general raising of awareness about climate change adaptation measures, local effects of climate change etc. Awareness raising focuses more explicitly on the communication of climate change: informing actors and of its importance while "assessing" focuses more on assessment of the potential effects of climate change for the purpose of developing suitable adaptation measures.
	Assessing	Assessing impacts, vulnerability, risks, and resilience: This first step consists of initial and periodically updated assessments, grounded in systematic observations and the best available science, of how climate change is impacting, and is expected to impact, natural and human systems. It also includes an assessment of the capacity of these systems to adapt to climate change impacts.
	Planning	Planning for adaptation: Subsequently, it is necessary to identify potential adaptation measures, and evaluate these measures – using, for example, a cost effectiveness analysis – to prioritize and choose among the options available. Adaptation planning should seek to avoid duplication, prevent maladaptation, and contribute to sustainable development outcomes. This step, along with the following implementation step, is undertaken while acknowledging that, in some cases, not all impacts will be addressed by planned adaptation, and there will be a role for contingency arrangements and loss acceptance.
	Financing of measures	Bearing the cost of adaptation measures, compensating the damages inflicted by climate impacts or adaptation measures
	Implementing	Implementing adaptation measures: Once a plan is in place, the next step is to implement adaptation initiatives at various levels – including local, national, and regional – and through a variety of means, including projects, programmes, policies, or strategies. Increasingly, adaptation is pursued through mainstreaming initiatives into budgets, sectoral policies, and sustainable development plans, though efforts can nonetheless be undertaken as stand-alone processes.
	Monitoring and evaluating	Monitoring and evaluating (M&E) adaptation: Finally, a continuous process of monitoring and evaluating adaptation is undertaken. This helps ensure that efforts are successful and that the knowledge and information generated from planning and implementation are fed back into the process to bolster the success of future efforts. Monitoring seeks to keep a record of progress made throughout the implementation phase, while evaluation seeks to determine the effectiveness of the initiative.

3. Actor-roles analysis

Table 2 includes the results of the analysis of relationships between actors and specific roles. We performed a chi-squared test of independence. The table shows expected versus observed frequencies of actor/role combinations.

Table 2. Actors and their roles in adapting to climate change. expected versus observed *frequencies of actor/role combinations.*

Actor	Role: Assessing	Awareness raising	Coordinating interaction	Financing of measures	Implementing	Monitoring and evaluating	Other	Planning	***Total***
Academia	37<br>9<br>30.8 %<br>26.4 %	26<br>16<br>21.7 %<br>10.9 %	7<br>11<br>5.8 %<br>4.2 %	0<br>22<br>0 %<br>0 %	10<br>38<br>8.3 %<br>1.8 %	23<br>6<br>19.2 %<br>27.7 %	1<br>1<br>0.8 %<br>6.7 %	16<br>17<br>13.3 %<br>6.3 %	120<br>120<br>100 %<br>6.7 %
Civil society (international/multinational/national)	3<br>8<br>2.9 %<br>2.1 %	19<br>14<br>18.1 %<br>8 %	16<br>10<br>15.2 %<br>9.6 %	26<br>19<br>24.8 %<br>7.9 %	28<br>33<br>26.7 %<br>5 %	2<br>5<br>1.9 %<br>2.4 %	2<br>1<br>1.9 %<br>13.3 %	9<br>15<br>8.6 %<br>3.5 %	105<br>105<br>100 %<br>5.9 %
Civil society (sub-national or local)	9<br>20<br>3.6 %<br>6.4 %	50<br>33<br>20 %<br>21 %	44<br>23<br>17.6 %<br>26.3 %	35<br>46<br>14 %<br>10.6 %	77<br>78<br>30.8 %<br>13.8 %	7<br>12<br>2.8 %<br>8.4 %	1<br>2<br>0.4 %<br>6.7 %	27<br>36<br>10.8 %<br>10.6 %	250<br>250<br>100 %<br>14 %
Government (local)	34<br>28<br>9.4 %<br>24.3 %	40<br>48<br>11.1 %<br>16.8 %	40<br>34<br>11.1 %<br>24 %	57<br>66<br>15.8 %<br>17.3 %	86<br>113<br>23.9 %<br>15.4 %	20<br>17<br>5.6 %<br>24.1 %	1<br>3<br>0.3 %<br>6.7 %	82<br>51<br>22.8 %<br>32.2 %	360<br>360<br>100 %<br>20.1 %
Government (national)	19<br>21<br>7 %<br>13.6 %	32<br>36<br>11.8 %<br>13.4 %	19<br>25<br>7 %<br>11.4 %	76<br>50<br>28 %<br>23.1 %	65<br>85<br>24 %<br>11.6 %	10<br>13<br>3.7 %<br>12 %	3<br>2<br>1.1 %<br>20 %	47<br>39<br>17.3 %<br>18.4 %	271<br>271<br>100 %<br>15.2 %
Government (sub-national)	19<br>15<br>9.8 %<br>13.6 %	23<br>26<br>11.9 %<br>9.7 %	22<br>18<br>11.3 %<br>13.2 %	32<br>36<br>16.5 %<br>9.7 %	47<br>61<br>24.2 %<br>8.4 %	11<br>9<br>5.7 %<br>13.3 %	1<br>2<br>0.5 %<br>6.7 %	39<br>28<br>20.1 %<br>15.3 %	194<br>194<br>100 %<br>10.9 %
Individuals or households	4<br>19<br>1.6 %<br>2.9 %	10<br>33<br>4.1 %<br>4.2 %	13<br>23<br>5.3 %<br>7.8 %	21<br>45<br>8.6 %<br>6.4 %	175<br>77<br>71.4 %<br>31.2 %	5<br>11<br>2 %<br>6 %	2<br>2<br>0.8 %<br>13.3 %	15<br>35<br>6.1 %<br>5.9 %	245<br>245<br>100 %<br>13.7 %
International or multinational governance institutions	5<br>6<br>6.9 %<br>3.6 %	13<br>10<br>18.1 %<br>5.5 %	3<br>7<br>4.2 %<br>1.8 %	36<br>13<br>50 %<br>10.9 %	8<br>23<br>11.1 %<br>1.4 %	1<br>3<br>1.4 %<br>1.2 %	2<br>1<br>2.8 %<br>13.3 %	4<br>10<br>5.6 %<br>1.6 %	72<br>72<br>100 %<br>4 %
Other	2<br>3<br>6.1 %<br>1.4 %	13<br>4<br>39.4 %<br>5.5 %	1<br>3<br>3 %<br>0.6 %	4<br>6<br>12.1 %<br>1.2 %	8<br>10<br>24.2 %<br>1.4 %	0<br>2<br>0 %<br>0 %	0<br>0<br>0 %<br>0 %	5<br>5<br>15.2 %<br>2 %	33<br>33<br>100 %<br>1.8 %
Private sector (corporations)	3<br>3<br>6.8 %<br>2.1 %	5<br>6<br>11.4 %<br>2.1 %	1<br>4<br>2.3 %<br>0.6 %	14<br>8<br>31.8 %<br>4.3 %	15<br>14<br>34.1 %<br>2.7 %	2<br>2<br>4.5 %<br>2.4 %	1<br>0<br>2.3 %<br>6.7 %	3<br>6<br>6.8 %<br>1.2 %	44<br>44<br>100 %<br>2.5 %
Private sector (SME)	5<br>7<br>5.4 %<br>3.6 %	7<br>12<br>7.5 %<br>2.9 %	1<br>9<br>1.1 %<br>0.6 %	28<br>17<br>30.1 %<br>8.5 %	41<br>29<br>44.1 %<br>7.3 %	2<br>4<br>2.2 %<br>2.4 %	1<br>1<br>1.1 %<br>6.7 %	8<br>13<br>8.6 %<br>3.1 %	93<br>93<br>100 %<br>5.2 %
Total	140<br>140<br>7.8 %<br>100 %	238<br>238<br>13.3 %<br>100 %	167<br>167<br>9.3 %<br>100 %	329<br>329<br>18.4 %<br>100 %	560<br>560<br>31.3 %<br>100 %	83<br>83<br>4.6 %<br>100 %	15<br>15<br>0.8 %<br>100 %	255<br>255<br>14.3 %<br>100 %	1787<br>1787<br>100 %<br>100 %

χ^2=610.772 · df=70 · Cramer's V=0.221 · Fisher's p=0.000

observed values
expected values
% within Actor
% within Role

4. Actor-roles analysis per region and settlement type

Table 3 includes an overview of the frequencies of observations of actor-role combinations per region. Figure 3 shows the relative frequencies of actor-role combinations in an aggregated form for the different settlement types.

Table 3: Number of observed actor-role combinations per region.

Region	Actor	Awareness raising	Assessing	Planning	Financing	Implementing	Coordinating interaction	Monitoring and evaluating	Other
Africa	Individuals or households	14	14	37	56	320	27	5	5
Africa	Civil society (sub-national or local)	21	6	11	11	30	19	3	0
Africa	Civil society (international/multinational/national)	9	1	4	14	16	7	1	0
Africa	Government (local)	15	9	24	13	23	9	3	2
Africa	Government (sub-national)	4	1	2	5	7	1	0	1
Africa	Government (national)	30	13	27	43	42	12	7	1
Africa	International or multinational governance institutions	2	3	8	23	9	2	5	0
Africa	Private sector (SME)	3	0	5	18	29	2	1	1
Africa	Private sector (corporations)	1	0	2	6	8	0	0	0
Africa	Academia	8	12	2	0	5	3	9	0
Africa	Other	6	1	2	2	2	0	0	0
Asia	Individuals or households	20	13	44	65	320	22	3	4
Asia	Civil society (sub-national or local)	22	4	9	19	35	30	5	1
Asia	Civil society (international/multinational/national)	11	1	1	11	11	6	0	0
Asia	Government (local)	23	12	27	37	43	18	8	0
Asia	Government (sub-national)	11	4	13	18	13	5	3	0
Asia	Government (national)	29	16	53	52	52	20	5	2
Asia	International or multinational governance institutions	9	3	2	22	6	6	0	0
Asia	Private sector (SME)	0	1	2	21	33	2	1	0
Asia	Private sector (corporations)	4	0	1	11	7	1	1	0
Asia	Academia	7	8	4	0	7	3	8	0
Asia	Other	5	0	0	1	2	0	0	0
Australasia	Individuals or households	4	4	7	5	28	2	3	0
Australasia	Civil society (sub-national or local)	3	1	1	1	6	5	0	0
Australasia	Civil society (international/multinational/national)	1	0	0	0	1	0	0	0
Australasia	Government (local)	4	6	9	4	6	7	0	0
Australasia	Government (sub-national)	2	3	5	1	2	2	0	0
Australasia	Government (national)	3	3	6	7	5	1	1	0
Australasia	International or multinational governance institutions	1	0	0	2	1	0	0	0
Australasia	Private sector (SME)	1	1	2	7	9	1	0	1
Australasia	Private sector (corporations)	1	0	1	4	4	0	1	0
Australasia	Academia	1	3	1	0	0	0	1	0
Australasia	Other	0	1	0	2	1	0	0	0

Central and South America	Individuals or households	5	1	7	5	36	3	0	0
Central and South America	Civil society (sub-national or local)	8	2	6	6	9	11	2	0
Central and South America	Civil society (international/multinational/national)	0	0	1	1	0	1	1	0
Central and South America	Government (local)	3	2	5	3	4	4	1	0
Central and South America	Government (sub-national)	2	0	3	2	3	2	0	0
Central and South America	Government (national)	5	3	7	9	9	5	0	0
Central and South America	International or multinational governance institutions	2	0	1	6	2	1	1	0
Central and South America	Private sector (SME)	1	0	0	2	3	0	1	0
Central and South America	Private sector (corporations)	0	0	0	1	0	0	0	0
Central and South America	Academia	3	3	2	0	0	1	1	1
Central and South America	Other	1	1	2	1	2	1	0	0
Europe	Individuals or households	4	1	8	12	43	1	0	0
Europe	Civil society (sub-national or local)	3	0	3	1	4	3	0	0
Europe	Civil society (international/multinational/national)	1	0	0	0	1	1	0	0
Europe	Government (local)	2	7	27	14	26	6	3	0
Europe	Government (sub-national)	4	4	13	3	7	4	1	0
Europe	Government (national)	8	11	23	16	18	7	2	0
Europe	International or multinational governance institutions	2	0	1	3	1	1	0	1
Europe	Private sector (SME)	5	2	6	9	16	1	3	0
Europe	Private sector (corporations)	2	1	3	5	5	1	1	0
Europe	Academia	4	8	1	0	2	1	2	0
Europe	Other	1	0	0	0	0	0	0	0
North America	Individuals or households	1	5	9	7	56	4	2	0
North America	Civil society (sub-national or local)	7	1	2	4	12	7	3	1
North America	Civil society (international/multinational/national)	1	0	0	1	1	1	0	0
North America	Government (local)	7	15	27	10	25	5	8	0
North America	Government (sub-national)	7	9	17	9	21	8	6	1
North America	Government (national)	8	11	11	19	16	6	5	1
North America	International or multinational governance institutions	0	1	1	1	1	1	1	0
North America	Private sector (SME)	2	2	7	7	18	2	2	0
North America	Private sector (corporations)	1	0	0	3	4	1	1	0
North America	Academia	7	12	3	0	2	3	3	0
North America	Other	5	2	2	0	3	4	1	0
Small Islands	Individuals or households	4	0	1	2	15	1	0	2
Small Islands	Civil society (sub-national or local)	3	0	2	2	4	1	0	0
Small Islands	Civil society (international/multinational/national)	2	0	1	4	0	2	0	1
Small Islands	Government (local)	0	0	0	1	0	0	0	0
Small Islands	Government (sub-national)	0	1	1	0	2	1	0	0

Small Islands	Government (national)	3	2	7	5	7	3	0	1
Small Islands	International or multinational governance institutions	2	0	2	5	0	1	0	1
Small Islands	Private sector (SME)	0	0	0	0	0	0	0	0
Small Islands	Private sector (corporations)	0	1	0	1	2	0	0	0
Small Islands	Academia	0	1	0	0	0	0	1	0
Small Islands	Other	1	0	0	0	0	0	0	0

Actors (aggregated) per settlement type by region

urban

	Africa	Asia	Australasia	Central and South America	Europe	North America	Small Island States
Individuals or households	26.37	23.36	16.36	14	12	13.75	15.38
Civil society	17.58	18.98	14.55	22	9	11.25	30.77
Government	35.16	38.69	50.91	40	56	55	38.46
International or multinational governance institutions	7.69	6.57	1.82	10	3	2.5	7.69
Private sector	7.69	7.3	10.91	4	10	7.5	7.69
Academia	4.4	1.46	0	6	8	5	0
Other	1.1	3.65	5.45	4	2	5	0

ambiguous

	Africa	Asia	Australasia	Central and South America	Europe	North America	Small Island States
Individuals or households	30.83	18.75	22.22	13.04	20.69	18.92	16
Civil society	10.53	14.06	22.22	10.87	5.17	8.11	22
Government	31.58	40.63	24.44	45.65	49.14	42.34	30
International or multinational governance institutions	13.53	10.94	13.33	19.57	6.9	0.9	22
Private sector	10.53	10.16	13.33	6.52	15.52	16.22	4
Academia	2.26	4.69	4.44	4.35	1.72	10.81	6
Other	0.75	0.78	0	0	0.86	2.7	0

rural

	Africa	Asia	Australasia	Central and South America	Europe	North America	Small Island States
Individuals or households	50.83	49.75	41.51	39.25	41.18	36.64	37.5
Civil society	11.6	10.87	11.32	18.69	11.76	11.45	17.5
Government	21.73	26.32	24.53	25.23	25.49	32.06	32.5
International or multinational governance institutions	3.31	2.89	1.89	4.67	0	2.29	5
Private sector	7.18	6.28	15.09	4.67	19.61	12.21	5
Academia	3.68	3.4	3.77	4.67	1.96	3.82	0
Other	1.66	0.51	1.89	2.8	0	1.53	2.5

Created with Datawrapper

Figure 3: Relative frequencies of actor-role combinations in an aggregated form for the different settlement types.

5. Transformational adaptation analysis

As an indicator for potentially transformational adaptation, we use the variable 'depth' that was coded in the GAMI database. GAMI operationalises transformation according to the dimensions depth, scope, speed, and limits, building on [6]. The definition behind the variable 'depth' aligns

closest with the IPCC definition of transformational adaptation[1], which we adopt. In GAMI, “the depth of a response relates to the degree to which a change reflects something new, novel, and different from existing norms and practices. A change that has limited depth would follow business-as-usual practices, with no real difference in the underlying values, assumptions and norms. This would include responses that are largely based on expansion of existing practices rather than consideration of entirely new practices. In-depth change, in contrast, might involve radically changing practices by altering frames, values, logics, and assumptions underlying the system. This might involve deep structural reform, complete change in mindset by governments or populations, radical shifts in public perceptions or values, and changing institutional or behavioural norms.”[8]

Accordingly, evidence of ‘depth’ of adaptation is operationalized into low, medium, and high levels (Table 4).

Table 4: Operationalisation of “depth” in evidence on adaptation.[3]

Low	Medium	High
Adaptations are largely expansions of existing practices, with minimal change in underlying values, assumptions or norms.	Adaptations reflect a shift away from existing practices, norms or structures to some extent.	Adaptations reflect entirely new practices that involve deep structural reform, complete change in mindset, major shifts in perceptions or values and changing institutional or behavioural norms.

Table 5 shows the full results of the ordered logistic regression analysis of low versus medium depth or medium versus high depth when there is a given variable.

Table 5: Characteristics of medium-to-high-depth responses in relation to the variables actor type, role, response type, region, and settlement type. Statistically significant ($p < 0.05$) coefficients of the ordered logistic regression predict depth levels. The coefficients are the log odds of low versus medium depth or medium versus high depth when a given variable exists. Studies that mention Small Island States, for instance, have a 3.7 (exp(1.3)=3.7) times higher likelihood of being associated with higher depth levels than studies that do not mention Small Island States.

	Value	Std. Error	t value	p value
**Africa**	-0.113	0.199	-0.568	0.570
**Asia**	0.061	0.188	0.327	0.743
**Australasia**	-0.021	0.311	-0.068	0.946
**Central and South America**	0.202	0.267	0.756	0.450
**Europe**	-0.387	0.269	-1.437	0.151
**North America**	0.430	0.230	1.867	0.062
**Small Island States**	1.308	0.401	3.266	0.001
**Behavioural/cultural**	-0.651	0.180	-3.622	0.000
**Ecosystem-based**	0.055	0.156	0.349	0.727
**Institutional**	0.553	0.161	3.424	0.001
**Technological/infrastructural**	0.243	0.162	1.501	0.133
**settlement_ambiguous**	0.372	0.710	0.524	0.600
**settlement_rural**	0.972	0.664	1.464	0.143

[1] “Adaptation that changes the fundamental attributes of a social-ecological system in anticipation of climate change and its impacts” [7].

**settlement_unclear**	0.408	0.685	0.595	0.552
**settlement_urban**	0.482	0.674	0.714	0.475
**Academia**	0.526	0.286	1.838	0.066
**Civil society (international/multinational/national)**	0.214	0.297	0.723	0.470
**Civil society (sub-national or local)**	-0.072	0.197	-0.362	0.717
**Government (local)**	0.353	0.198	1.788	0.074
**Government (national)**	0.118	0.176	0.669	0.504
**Government (sub-national)**	0.352	0.233	1.512	0.131
**Individuals or households**	-0.201	0.213	-0.943	0.346
**International or multinational governance institutions**	0.569	0.269	2.116	0.034
**Other**	0.276	0.406	0.680	0.496
**Private sector (corporations)**	0.466	0.335	1.393	0.163
**Private sector (SME)**	-0.071	0.245	-0.292	0.770
**Assessing**	-0.079	0.197	-0.399	0.690
**Awareness raising**	0.079	0.173	0.456	0.649
**Coordinating interaction**	0.392	0.185	2.124	0.034
**Financing of measures**	-0.111	0.169	-0.656	0.512
**Implementing**	-0.285	0.350	-0.812	0.417
**Monitoring and evaluating**	-0.736	0.260	-2.834	0.005
**Other**	0.436	0.441	0.989	0.323
**Planning**	0.217	0.167	1.300	0.193
**low|medium**	1.787	0.785	2.276	0.023
**medium|high**	4.991	0.820	6.085	0.000

6. References

1. Dodman, D. *et al.* Cities, settlements and key infrastructure. in *Climate Change 2022: Impacts, Adaptation and Vulnerability. Contribution of Working Group II to the Sixth Assessment Report of the Intergovernmental Panel on Climate Change* (eds. Pörtner, H.-O. et al.) 907–1040 (Cambridge University Press, 2022). doi:10.1017/9781009325844.008.
2. Satterthwaite, D. Background Paper: Small and intermediate urban centres in sub---Saharan Africa. *UrbanARK Working Paper* **6**, (2016).
3. Berrang-Ford, L. *et al.* A systematic global stocktake of evidence on human adaptation to climate change. *Nat. Clim. Chang.* 989–1000 (2021) doi:10.1038/s41558-021-01170-y.
4. UNCC. *25 Years of Adaptation under the UNFCCC*. (2019).
5. Mees, H., Driessen, P. P. & Runhaar, H. A. Exploring the scope of public and private responsibilities for climate adaptation. *Journal of environmental policy & planning* **14**, 305–330 (2012).
6. Termeer, C. J. A. M., Dewulf, A. & Biesbroek, G. R. Transformational change: governance interventions for climate change adaptation from a continuous change perspective. *Journal of Environmental Planning and Management* **60**, 558–576 (2017).
7. IPCC. Annex II: Glossary. in *Climate Change 2022: Impacts, Adaptation, and Vulnerability. Contribution of Working Group II to the Sixth Assessment Report of the Intergovernmental Panel on Climate Change* (eds. Pörtner, H. O. et al.) (Cambridge University Press, 2022).
8. Lesnikowski, A. *et al. The Global Adaptation Mapping Initiative (GAMI): Part 3 – Coding protocol*. https://protocolexchange.researchsquare.com/article/pex-1242/v1 (2021) doi:10.21203/rs.3.pex-1242/v1.
